# Embodied metacognition as strengthened functional connection between neural correlates of metacognition and dance in dancers: exploring creativity implications

**DOI:** 10.3389/fnhum.2024.1347386

**Published:** 2024-02-15

**Authors:** Ching-Ju Yang, Hsin-Yen Yu, Tzu-Yi Hong, Li-Kai Cheng, Wei-Chi Li, Tzu-Chen Yeh, Li-Fen Chen, Jen-Chuen Hsieh

**Affiliations:** ^1^Institute of Brain Science, College of Medicine, National Yang Ming Chiao Tung University, Taipei, Taiwan; ^2^Integrated Brain Research Unit, Division of Clinical Research, Department of Medical Research, Taipei Veterans General Hospital, Taipei, Taiwan; ^3^Graduate Institute of Arts and Humanities Education, Taipei National University of the Arts, Taipei, Taiwan; ^4^Center for Intelligent Drug Systems and Smart Bio-devices (IDS^2^B), National Yang Ming Chiao Tung University, Hsinchu, Taiwan; ^5^Department of Biological Science and Technology, College of Biological Science and Technology, National Yang Ming Chiao Tung University, Hsinchu, Taiwan; ^6^Department of Radiology, Taipei Veterans General Hospital, Taipei, Taiwan; ^7^Institute of Biomedical Informatics, College of Medicine, National Yang Ming Chiao Tung University, Taipei, Taiwan; ^8^Brain Research Center, National Yang Ming Chiao Tung University, Taipei, Taiwan

**Keywords:** dancer, metacognition, creativity, originality, flexibility, motor, functional connectivity, resting-state functional MRI

## Abstract

**Introduction:**

Dance education fosters embodied metacognition, enhancing student’s creativity. This study examines the crucial role of functional connectivity (FC) between the neural correlates of metacognition (NCM) and dance (NCD) as the neurological foundation for dancers’ embodied metacognition. The investigation also explores whether these consolidated FCs inform the general creativity in dancers.

**Methods:**

The research involved 29 dancers and 28 non-dancer controls. The study examined resting-state connections of the NCM through seed-based FC analysis. Correlation analyses were employed to investigate the connections between the targeted NCM-NCD FCs, initiated from the *a priori* NCM seed, and general creativity.

**Results:**

Dancers demonstrated heightened FC between NCM and NCD compared to non-dancer controls. The targeted regions included the putamen, globus pallidus, posterior cerebellum, and anterior insula of NCD. The dancers exhibited higher originality scores. In dancers, the enhanced FC showed a negative correlation with originality and a positive correlation with flexibility. Conversely, the controls exhibited no significant correlations.

**Discussion:**

Extended dance training enhances the NCM-NCD connection signifying embodied metacognition. This interconnectedness may serve as the neural predisposition for fostering general creativity performance in dancers. Dancers with heightened levels of originality could leverage the relatively weaker NCM-NCD FCs to facilitate better integration and coordination of creative cognitive processes. Our findings suggest that the consolidated functional connections as sculpted by domain-specific training may inform general creativity.

## 1 Introduction

Metacognition, the reflective examination of cognitive processes (e.g., memory, perception, and judgment), consists primarily of three interconnected components—metacognitive knowledge, metacognitive experience, and metacognitive monitoring and control (Flavell, 1979). Metacognition is crucial in art education, particularly in dance (Douglas, 2017; Buck-Pavlick, 2022). In dance, embodied metacognition involves a profound understanding of cognitive processes within the realm of bodily movement and expression. Dancers leverage this awareness to optimize performance and skill development by strategically managing attention in areas like muscle engagement, fellow dancers’ positions, memorized movements, and emotional portrayals (van Vugt, 2014). This extends beyond conventional metacognition, involving the intricate interplay between physical sensations, motor control, and cognitive reflections during dance (Moffett, 2012; van Vugt, 2014). Dancers practicing embodied metacognition attune themselves to their body’s signals, continually refining movements based on introspective insights (van Vugt, 2014; Christensen et al., 2018). Active metacognition empowers dancers, enhancing performance quality and forging a deep connection between cognitive awareness and dance artistry (Cooper, 2013). This engagement facilitates rapid progress and continuous refinement, infusing movements with authenticity and meaningful expression (Cooper, 2013; MacIntyre et al., 2014). While our comprehension of embodied metacognition in dancers has advanced, the neurological representation of embodied metacognition remains elusive.

Creativity in dance relies on embodied metacognition, involving cognitive knowledge and regulation (May et al., 2011, 2020; Hanna, 2014). Dancers tap into creativity by reflecting on personal experiences and emotions. Research highlights the influence of metacognitive elements—knowledge, experience, monitoring, and control—on creativity (Fayena-Tawil et al., 2011; Lizarraga and Baquedano, 2013; Jia et al., 2019). Metacognition supports diverse aspects of dance creativity, such as movement creation, interoceptive awareness, self-reflection, risk-taking, adaptability, emotional expression, problem-solving, artistic clarity, collaboration, continuous learning, and inspiration (May et al., 2011, 2020; Cooper, 2013; Hanna, 2014; Christensen et al., 2018). Mental training for embodied metacognition, particularly through validated use of mental imagery, enhances both specific choreographic creativity and general creativity in dance students (May et al., 2020). Kaufman and Beghetto (2009) developed a framework categorizing creativity into four distinct levels: mini-C, which encompasses personal insights; little-C, referring to everyday creativity recognized by non-experts; Pro-C, denoting professional contributions in a domain that are acknowledged by experts, typically following extensive practice; and Big-C, which represents eminent creativity that has a significant impact on culture. According to this model, progression through these stages isn’t a prerequisite for reaching eminent creativity (Preiss, 2022). Dancers who have undergone extensive professional training and gained substantial experience can likely achieve Pro-C status, integrating their dance expertise with metacognitive knowledge. This integration enhances the precision of evaluation and judgment in dance performance, contributing to aesthetically pleasing expressions. However, the impacts of embodied metacognition on general creativity and its neurological underpinnings in dancers are still largely unknown.

Neural correlates of metacognition (NCM), involving the lateral and medial prefrontal cortex (PFC), insula/inferior frontal gyrus (IFG), dorsal anterior cingulate cortex/pre-supplementary motor area (dACC/pre-SMA), precuneus, and ventral striatum, plays a pivotal role in various metacognition-related cognitive domains, including memory, perception, and decision-making (Fleming and Dolan, 2012; Morales et al., 2018; Vaccaro and Fleming, 2018). The neural correlates of dance (NCD) involve brain regions that process the motor, cognitive, emotional, spatial, temporal, and bodily dimensions of dance during performance, perception, imagination, and creation (Sevdalis and Keller, 2011; Bläsing et al., 2012; Karpati et al., 2015; Savrami, 2017; Basso et al., 2020; Zardi et al., 2021; Foster Vander Elst et al., 2023; Yang et al., 2023). NCD’s motor components, linked to dance-related motor learning, involve the motor cortices, premotor cortex, supplementary motor area (SMA), basal ganglia, and cerebellum (Hänggi et al., 2010; Karpati et al., 2015; Li et al., 2015; Lu et al., 2018; Basso et al., 2020; Foster Vander Elst et al., 2023; Yang et al., 2023). Non-motor components of NCD, linked to cognitive and socio-affective dimensions of dance, include the insula, frontoparietal regions (mirror neuron network/action observation network), superior temporal gyrus/superior temporal sulcus (STG/STS), and limbic system substrates (Karpati et al., 2015; Burzynska et al., 2017; Zardi et al., 2021; Foster Vander Elst et al., 2023). Long-term artistic training may consolidate relevant networks and functional connections in the resting brain (Lin et al., 2013; Cheng et al., 2023; Hong et al., 2023a,b; Yang et al., 2023). Acknowledging the crucial role of metacognition in dance training, our proposition asserts that proficient dancers are likely to display enhanced connectivity between NCM and NCD, especially in motor components. This increased connectivity serves as a neural marker indicative of embodied metacognition. Subsequent analyses provide supporting evidence for the predilection influence of the NCM-NCD connection on the overall general creativity of dancers (cf., Yang et al., 2023).

## 2 Materials and methods

### 2.1 Participants

Right-handed participants in this study were recruited from individuals majoring in dance (DANCE) and non-dancer controls (CON). After 14 participants chose to withdraw from the study and an additional 12 were excluded due to structural brain abnormalities, severe motion artifacts, or technical issues with data collection, a total of 29 DANCEs (mean age 23.1 ± 2.9 years) and 28 CONs (mean age 22.8 ± 1.6 years), carefully matched for age and education level, were included in the analyses. None of the participants in both the DANCE and CON groups reported having received any training in sports. All participants were selected from the identical sample previously detailed in our earlier study, and specifics regarding demographics and dance training can be found in Table 1 of the published work (Yang et al., 2023). The study received approval from the Institutional Review Board of Taipei Veterans General Hospital, and written informed consent was obtained from each participant.

### 2.2 Assessment of general creativity performance

The Abbreviated Torrance Test for Adults (ATTA) was employed to assess general creativity (Chen, 2006). The ATTA battery includes one verbal and two figural tests, with four norm-referenced creativity indicators (fluency, originality, elaboration, flexibility), a creativity index (the sum of the aforementioned 4 measures), and two criterion-referenced creativity indicators (verbal and visual creativity) calculated for an overall creativity profile of each participant (Kharkhurin and Samadpour Motalleebi, 2008; Althuizen et al., 2010; Kharkhurin, 2010; Shen and Lai, 2014; Sunavsky and Poppenk, 2020). A comparison of the creativity profiles between the DANCE and CON groups was conducted based on the six indicators of general creativity. Between-group differences were assessed using a two-sample *t*-test (SPSS Statistics version 27.0, SPSS Inc., USA), with statistical significance set at *p* < 0.05.

### 2.3 MRI data acquisition

Magnetic resonance imaging was conducted using the 3T MAGNETOM Trio™ system, with participants positioned supine within the scanner. To minimize motion artifacts, foam cushions were used for head fixation inside the head coil. Resting-state functional scans were obtained through a T2*-weighted gradient echo planar imaging (EPI) sequence with the following parameters: repetition time (TR) = 2500 ms, echo time (TE) = 30 ms, flip angle = 90°, field of view (FOV) = 220 × 220 mm^2^, slice thickness = 3.4 mm, slice number = 40, matrix size = 64 × 64, tilted angle = 30°, and voxel size = 3.4 mm × 3.4 mm × 3.4 mm. Each resting-state fMRI time series consisted of 200 volumes, with a duration of 500 s per time series. Additionally, T1-weighted structural images were acquired using the magnetization-prepared rapid gradient echo (MPRAGE) sequence with the following parameters: TR = 2530 ms, TE = 3.03 ms, flip angle = 7°, FOV = 224 × 256 mm^2^, matrix size = 224 × 256, and slice thickness = 1 mm. Participants were instructed to maintain a motionless and alert state, keeping their eyes open and refraining from engaging in any specific thoughts.

### 2.4 Data preprocessing

The advanced DPARSF module V5.4 was used to preprocess the resting-state fMRI data (Yan et al., 2016). The preprocessing involved a series of sequential steps, starting with slice timing correction and followed by realignment to correct for head motion. Participants displaying head motion exceeding 2 mm displacement or 2° rotation in any cardinal direction were excluded. Subsequently, T1-weighted images were co-registered to the mean functional image using intra-subject spatial alignment. The segmentation of gray matter, white matter, and cerebrospinal fluid was carried out using the unified segmentation model. Nuisance regression utilized the Friston 24-parameter model (Friston et al., 1996) and default masks from SPM, eliminating head motion parameters and signals from white matter and cerebrospinal fluid. Spatial normalization to a study-specific DARTEL template (Ashburner, 2007), transformed to MNI space, was performed with image resampling to 3 mm isotropic voxels. Spatial smoothing was applied using a Gaussian kernel with a full width at half-maximum (FWHM) of 6 mm. Temporal band-pass filtering (0.01−0.1 Hz) was implemented to minimize high-frequency noise and low-frequency drift. Global signal regression (GSR) was not applied due to its tendency to amplify negative correlations and distort between-group differences (Murphy et al., 2009; Weissenbacher et al., 2009; Saad et al., 2012).

### 2.5 Resting-state functional connectivity

Metacognition-related regions, including the rostrolateral PFC (rlPFC, BA10), dorsolateral PFC (dlPFC, BA46), dACC/pre-SMA (BA32), medial PFC (mPFC, BA10/32), insula/IFG (BA47), precuneus (BA7/23), and ventral striatum, were defined as seed regions of interest (ROIs) since they have been identified in various tasks-based fMRI studies (Fleming et al., 2012; McCurdy et al., 2013; Morales et al., 2018; Vaccaro and Fleming, 2018). These seed ROIs were constructed as twelve 10-mm radius spheres centered at MNI coordinates identified by Morales et al. (2018) and Vaccaro and Fleming (2018) (see Table 1 for details of ROIs). The creation of these spheres was executed using WFU Pickatlas 3.0.5 (Maldjian et al., 2003). Given that dancers dynamically engage different aspects of metacognitive functioning for their learning and performance, it’s logical to merge individual ROIs into a unified, overarching composite ROI for resting-state functional connectivity (FC) analysis. This approach is rooted in the belief that these dispersed regions, having interconnected functions, are likely to function in a synergistic and holistic way (Rasero et al., 2018). The reference time course was derived by averaging the time courses of all voxels within this composite ROI consisting of 12 predefined ROIs. The FC map was then generated by assessing Pearson’s correlation coefficients (*r*) between the reference time course and the time course of each voxel of the brain. The *r*-value of each voxel was transformed to a *z*-value using Fisher’s *r*-to-*z* transformation to normalize the distribution. Multiple regression analyses were conducted on all *z*-transformed FC maps for controlling the effects of age and sex. Between-group comparisons were examined using two-sample *t*-tests on FC maps, with significance set at peak-level thresholds *p* < 0.005 and *p* < 0.001, followed by cluster-level *p*_*FWE*_ < 0.05 in SPM.

**TABLE 1:** *A priori* metacognition regions of interest for seed-based functional connectivity analysis.

Study	Region of interest	Laterality	BA	MNI coordinates		
				***x***	***y***	***z***
Morales et al., 2018	rostrolateral prefrontal cortex (rlPFC)	L	10	−33	44	28
		R	10	27	53	25
	dorsal anterior cingulate cortex/ pre-supplementary motor area (dACC/pre-SMA)	L/R	32	0	17	46
	precuneus	L/R	23	0	−64	24
Vaccaro and Fleming, 2018	posterior medial frontal cortex (pMFC)	L/R	8/32	−2	30	38
	insula/inferior frontal gyrus (insula/IFG)	L	47	−36	28	−6
		R	47	44	16	0
	dorsolateral prefrontal cortex (dlPFC)	L	46	−50	24	28
	anterior dorsolateral prefrontal cortex (ant. dlPFC)	R	10	28	50	26
	ventromedial prefrontal cortex (vmPFC)	L/R	32	−2	44	−12
	dorsal precuneus	R	7	12	−66	54
	ventral striatum	R		10	8	−2

L, left; R, right; BA, Brodmann’s area; MNI, Montreal Neurological Institute.

### 2.6 Correlation analysis

This study aimed to explore the impact of the interconnectedness between NCM and NCD on dancers’ general creativity performance, evaluated through the ATTA test battery. Drawing from the findings of May et al. (2020), three creativity indicators—fluency, originality, and flexibility—which exhibited a notable increase in dance students following metacognitive skills training were probed. Regions displaying significant between-group differences (DANCE vs. CON) in NCM-seeded FCs were identified. Spherical ROIs, each centered at the coordinates of these significant regions with a radius of 5 mm, were generated. The *z*-values extracted from these spherical ROIs were then correlated with ATTA metrics. Statistical significance was set at *p* < 0.05. Further, to address multiple comparisons, a Bonferroni correction was applied by adjusting the *p*-value to 0.0166 (0.05 divided by 3), given the three measures (fluency, originality, and flexibility) under examination.

## 3 Results

### 3.1 Creativity outcomes

The DANCE group exhibited significantly elevated originality scores on the ATTA (DANCE: 17.17 ± 1.77, CON: 15.32 ± 2.51, *p* = 0.002), with no discernible between-group differences observed for fluency, elaboration, flexibility, visual creativity, verbal creativity, or creativity index. These findings are derived from the identical sample and results reported in our earlier study (Yang et al., 2023).

### 3.2 Heightened connectivity between NCM and NCD in dancers

**FIGURE 1:**
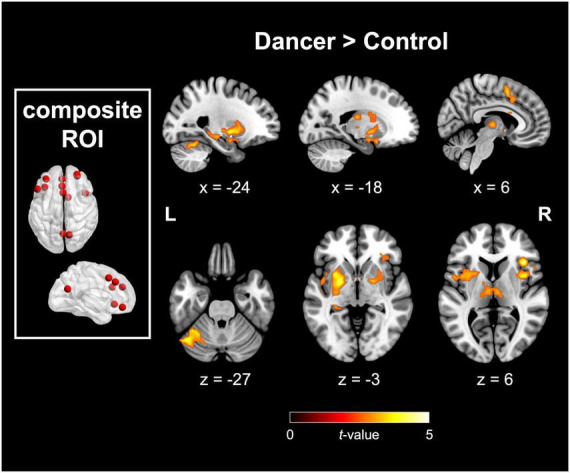
Between-group disparities in FC originating from the predefined composite metacognition mask. The composite metacognition ROI comprises regions associated with metacognition from Morales et al. (2018) and Vaccaro and Fleming (2018). Significant differences in FC, originating from the composite ROI, were noted between the dancer and control groups in both motor components of NCD (including the putamen, globus pallidus, supplementary motor area, cingulate motor area, and posterior cerebellum) and metacognition-related regions within the cingulo-opercular network (involving bilateral anterior insula, right inferior frontal gyrus, dorsal anterior cingulate cortex, and bilateral thalamus). FC, functional connectivity; ROI, region of interest; NCD, neural correlates of dance. Red color denotes the seed region and details for the coordinates are listed in Table 1. All displayed images are significant at a peak-level threshold *p* < 0.005, corrected for multiple comparisons at *p*_*FWE*_ < 0.05, with additional sub-significant findings at *p*_*FWE*_ = 0.061.

The DANCE group demonstrated elevated interconnectedness between NCM and NCD. The targeted motor components of NCD included the bilateral putamen, bilateral globus pallidus (GP), left posterior cerebellum (lobule VI and crus I), right SMA, and right dACC/cingulate motor area (CMA). Moreover, these target regions also covered non-motor components of NCD, such as the bilateral anterior insula (AI), right IFG, left hippocampus, left STG, left mediodorsal thalamus, and left amygdala. Figure 1 and Table 2 provide additional details.

**TABLE 2:** Between-group differences in functional connectivity seeded from *a priori* unified metacognition mask.

Contrast	Cluster-level	K	Region	Laterality	BA	MNI coordinates			*t-*value
	***p*_*FWE*_**					***x***	***y***	***z***	
*DANCE* > *CON*	<0.001	634	GP*	L		−21	−3	−3	4.36
			Putamen*	L		−27	6	−3	3.90
			Hippocampus	L		−27	−33	−6	3.59
			STG	L	22	−45	−15	0	3.55
			Thalamus (MD)	L		−3	−12	3	3.50
			AI*	L	13	−39	9	0	3.49
			Amygdala	L		−18	−3	−15	3.48
	0.007	204	IFG	R	45	39	21	6	4.68
			AI	R	13	39	6	9	3.92
			GP	R		18	0	−3	3.42
			Putamen	R		27	12	−6	3.12
	0.042	143	Cerebellar lobule VI*	L		−27	−57	−24	4.60
			Cerebellar crus I*	L		−42	−63	−27	4.28
	0.061	131	SMA	R	6	6	6	51	3.55
			dACC/CMA	R	24	9	12	36	3.55
*DANCE* < *CON*	NS								

Significant results at peak-level threshold *p* < 0.005, corrected for multiple comparisons at *p_*FWE*_* < 0.05, with additional sub-significant findings at *p*_*FWE*_ = 0.061. DANCE, dancer group; CON, control group; GP, globus pallidus; STG, superior temporal gyrus; MD, mediodorsal; AI, anterior insula, IFG, inferior frontal gyrus; SMA, supplementary motor area; dACC, dorsal anterior cingulate cortex; CMA, cingulate motor area; NS, not significant; also refer to Table 1 for other abbreviations. For more information, see Figure 1.

*Signifies statistical significance at peak-level threshold *p* < 0.001, followed by cluster-level *p*_*FWE*_ < 0.05.

### 3.3 Correlations between connectivity strength and behavioral variables

**FIGURE 2:**
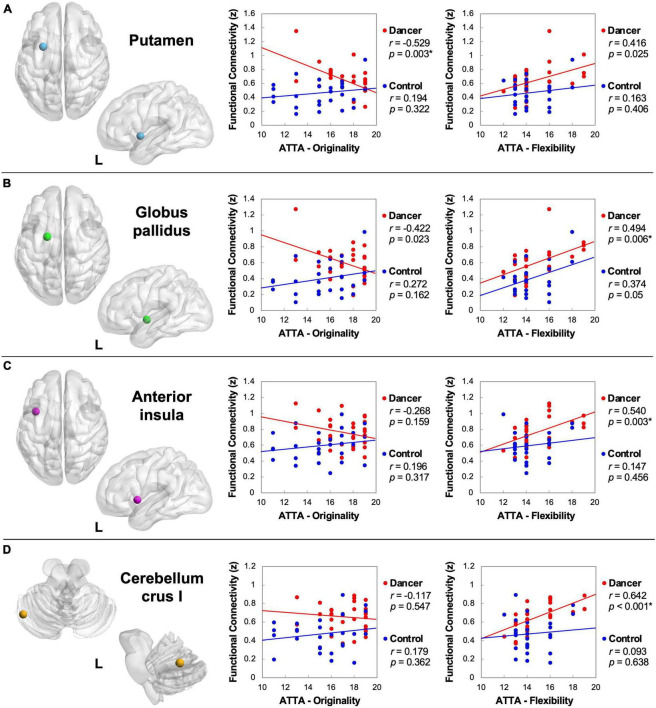
Abbreviated Torrance Test for Adults (ATTA) correlations with the strength of FC between NCM and NCD. Regarding ATTA originality, dancers demonstrate a discernible negative correlation between originality score and the strength of FC linking NCM and motor components of NCD [the putamen **(A)**]. Regarding ATTA flexibility, dancers demonstrate a discernible positive correlation between flexibility score and the strength of FC linking NCM and NCD [the globus pallidus **(B)**, anterior insula **(C)**, and cerebellar crus I **(D)**]. Collectively, the strength of NCM-NCD FC manifest a negative correlation trend with originality and a positive correlation trend with flexibility in dancers. These correlations are statistically non-significant in the control group. L, left; FC, functional connectivity; NCM, neural correlates of metacognition; NCD, neural correlates of dance. *Denotes significant results after Bonferroni correction (*p* < 0.0166).

The DANCE group demonstrated significant negative correlations between originality scores and the strength of FCs linking NCM with NCD, specifically the left putamen (*r* = −0.529, *p* = 0.003) and left GP (*r* = −0.422, *p* = 0.023) (Figures 2A, B). On the contrary, the DANCE group displayed distinct positive correlations between flexibility scores and the strength of FCs linking NCM and NCD, specifically the left putamen (*r* = 0.416, *p* = 0.025), left GP (*r* = 0.494, *p* = 0.006), and left cerebellar crus I (*r* = 0.642, *p* < 0.001) (Figures 2A, B, D). The left AI, a common neural substrate of NCM and NCD, was also targeted (*r* = 0.54, *p* = 0.003) (Figure 2C). Notably, the CON group exhibited no significant correlations in these aspects.

Upon detailed examination, within both the DANCE and CON groups, no substantial relationships were identified between the strength of NCM-NCD FCs and other ATTA metrics. These parameters encompass the creativity index, fluency, elaboration, as well as verbal and visual creativity metrics.

## 4 Discussion

Dancers showcased the expression of their embodied metacognition by exhibiting heightened interconnectedness between regions associated with metacognition and those linked to dance movements, motor imagery, spatial cognition, rhythm synchronization, salience detection, and emotional processing. Expanding on our prior investigations, the notable hyperconnectivity observed in areas related to motor functions reinforces and expands upon the notion that the neuroplastic changes associated with embodied learning in dancers are concentrated within the domain of dance movement. Furthermore, the correlations between FC strength and scores in originality and flexibility of the ATTA suggest that dancers achieve a harmonious blend of controlled and spontaneous creative cognition following extensive dance training.

### 4.1 Coalescence of NCM and NCD signifying embodied metacognition in dancers

In dancers, the NCM exhibit increased intrinsic FCs involving the AI, IFG, dACC/CMA, and rlPFC (Figure 1). Together with the mediodorsal thalamus, which is the target region of the extrinsic FC of NCM, all these regions collectively form the cingulo-opercular network, a key neural network involved in metacognition (Dosenbach et al., 2008; Morales et al., 2018). The cingulo-opercular network is recognized for its central role in the cognitive control of salience detection, reorientation, and mental switching, allowing for the flexible allocation of processing resources to other goal-relevant networks (i.e., the sensorimotor network) (Cocuzza et al., 2020). This neural network plays a significant role in fostering cognitive flexibility and participating in advanced cognitive functions such as attention, inhibition control, action preparation, working memory, and sensation (Camilleri et al., 2018). The AI and dACC/CMA have pivotal functions in detecting salience and integrating sensory, emotional, and cognitive information to foster self-awareness and social behavior (Craig, 2009; Menon and Uddin, 2010). The rlPFC empowers individuals to focus on environmental changes and on self-generated or sustained mental representations, often termed as “thoughts in our head” (Burgess et al., 2007). The enhanced intrinsic connectivity within the cingulo-opercular network may reflect the enhanced metacognitive abilities in dancers following extensive training.

In dance training, embodied metacognition demands that dancers comprehend dance concepts, infuse meaning into their movements, and apply their knowledge by deciding how to organize elements of body, gesture, locomotion, time, space, and energy (Hanna, 2014). Through extensive physical practice, rapid interactions are facilitated, allowing for quick adjustments in anticipation of performance outcomes. As expected, expert dancers demonstrated enhanced extrinsic FC between NCM and NCD (particularly motor components) as the neurological underpinning of (dancer) domain-specific embodied metacognition. This tight cognitive-dance movement interaction aligns with our earlier discoveries of optimized cortico-basal ganglia and cortico-cerebellar loops in dancers, particularly the interconnectedness of motor and cognitive/associative circuits (Yang et al., 2023). The specific regions targeted within the motor components of NCD encompassed both subcortical structures (putamen, GP, and posterior cerebellum) and cortical motor areas (SMA/CMA) (Figure 1). These motor substrates are acknowledged for their participation in executing movements (Haber, 2003; Errante and Fogassi, 2020). However, they play distinct roles in motor processing: the putamen regulates and facilitates voluntary movements, the GP inhibits movement, the posterior cerebellum coordinates movement and corrects prediction errors, and the SMA/CMA is involved in movement planning and anticipation (Hardwick et al., 2013; Koziol et al., 2014; Seghezzi et al., 2019; Rocha et al., 2023). The enhanced connectivity is consistent with the idea that dancers exercise heightened engagement in complex motor cognition, involving activities like nuanced action observation/simulation and refined motor imagery (Henschke and Pakan, 2023).

In dancers, the heightened FCs between NCM and NCD also involve the hippocampus, STG, and amygdala, as well as AI (the shared neural substrate of NCM and NCD) (Figure 1). These regions play roles in spatial cognition, rhythm synchronization, salience detection, and emotional processing. In the spatial dimension of dance, extensive training enhances dancers’ balance and spatial orientation skills, accompanied by observable increases in gray matter volumes in the hippocampus, insula, and CMA, setting dancers apart from non-dancers (Dordevic et al., 2018). Proficient dancers, in the temporal dimension of dance, exhibit heightened cortical thickness in the STG, particularly linked to rhythm synchronization, melody discrimination, and dance imitation, facilitating auditory–motor interaction in rhythmic contexts (Karpati et al., 2017). In the cognitive and emotional dimensions of dance, the AI serves a crucial integrative role, bridging physical, cognitive, and emotional domains, enabling advanced cognitive control and interoceptive awareness by converging various sensory and affective inputs (Craig, 2009, 2011). Both the AI and amygdala significantly contribute to processing socio-affective information, fostering emotional awareness and reactions associated with dancers’ empathic abilities (Gujing et al., 2019; Zardi et al., 2021). Alongside these two regions, the cerebellar crus I, in addition to participating in motor processing, plays a pivotal role in perception, emotion, and social cognition (Koziol et al., 2014; Baumann et al., 2015; Adamaszek et al., 2017; Van Overwalle et al., 2020). Overall, the increased connectivity may lead dancers to integrate top-down processes guided by knowledge with bottom-up processes driven by their dance experiences (Moffett, 2012).

The coordinated functioning of the identified areas, via both intrinsic and extrinsic connections in the NCM and NCD, could underpin the neural framework for dancers’ embodied metacognition. This coordination may heighten their metacognitive awareness and potentially improve their artistic expression in dance.

### 4.2 Creative originality and flexibility of dancers

#### 4.2.1 Enhanced originality in dancers

Metacognitive skills enable dancers to evaluate, adjust, and effectively apply their understanding and imagination to their physical movements. Dancers trained under special education system extensively utilize mental imagery and decision-making to execute movements as per a choreographer’s directives, processes that significantly depend on their metacognitive abilities (i.e., self-awareness and self-regulation) (May et al., 2011, 2020). Both (dance) domain-specific creativity and general creativity (specifically originality aspect) can be enhanced by honing metacognitive skills through mental imagery (May et al., 2020). The basal ganglia (putamen and GP) and cerebellum of NCD as identified in our study (Figure 1) subserve motor execution and motor imagery in creativity-related tasks (i.e., creative production) (Jeannerod, 1994; May et al., 2011; Brown and Kim, 2021; Zardi et al., 2021) and may play an important role in these cognitive-motor interactions (Leisman et al., 2014). Our study, while not measuring individual creativity in dance specifically, suggests that expert participants likely reached the Pro-C level due to their extensive training in specialized art education system. The findings from the ATTA indicate that the DANCE group demonstrated significantly enhanced performance in general creative originality, indicative of a successful influence from the Pro-C level of domain-specific creativity (Preiss, 2022).

#### 4.2.2 Absence of flexibility enhancement in dancers

In the realm of behavior, training that is tailored specifically to dance uniquely influences dancers’ ATTA performance. This impact manifests without significant variations in ATTA metrics, with the notable exception of originality. This observation aligns with research findings which suggest that metacognitive skills tailored, respectively, to different art forms may result in different outcomes of creativity aspects: dance imagery-based metacognition training predominantly fosters originality in dance students (May et al., 2020), while visual art-related metacognition training primarily promotes flexibility and fluency in students, but not originality (van de Kamp et al., 2015). Such a context-dependent distinction emphasizes the complex influence of metacognition on different aspects of domain-specific and domain-general creativity across various artistic disciplines. Therefore, we surmise that the specific nature of dance training, coupled with the verbal and visual format of the ATTA assessment, may contribute to the absence of change in ATTA flexibility and other metrics in dancers.

#### 4.2.3 Neural strategies for creativity in dancers

The observed diverging trends in how creative originality and flexibility dynamically correlate with the strength of NCM-NCD FCs suggest complex cognitive processes and neural strategies in dancers’ creativity. Albeit the absence of flexibility enhancement, the presence of significant positive correlations between the strength of NCM-NCD FCs and the ATTA flexibility scores suggests that the consolidated NCM-NCD FCs as sculpted by domain-specific training may inform the general creative flexibility performance in dancers (Figure 2).

The identified negative correlation between ATTA originality scores and the strength of NCM-NCD (the putamen and GP) FCs (Figures 2A, B) in dancers suggests that the loosening of the NCM-NCD bond may serve as a trait neural predisposition to strike a balance between metacognitive monitoring (for appropriateness or fit) and mind-wandering (for originality) during the creative process (Preiss, 2022). The putamen is primarily involved in initiating and regulating learned movement sequences, more so than in untrained movements (Pinsard et al., 2019). Similarly, the GP is a major output nucleus of the basal ganglia, helping regulate learned movement sequences and inhibit competing ones (Poldrack et al., 2005; Ashby et al., 2010). Both two regions contribute to habit learning and automaticity, which could restrict originality in creative performance (Ashby et al., 2010). The proposition that over-monitoring impedes originality offers a plausible explanation for the observed dissociation in the NCM-NCD connection among dancers with higher originality. The pursuit of equilibrium between creative originality and metacognitive monitoring emerges as a critical consideration for expert dancers. Our findings align with the concept articulated by Koestler (1964), viewing creativity as a process where originality triumphs over habitual behavior.

### 4.3 Limitations and future directions

In this study, we substantiated the connections between NCM-NCD FCs and the general creativity performances in dancers, employing the ATTA. However, there are points for further consideration. Focusing on neuroplasticity in dancers and requiring group comparisons, we used a well-established psychometric creativity test more aligned with our goals, allowing us to examine creativity’s cross-domain effects in dancers. Since specialized experience, as seen in choreography and movement creativity, plays a role in both general and domain-specific creativity (Hong and Milgram, 2010; Qian et al., 2019), more detailed behavioral studies are needed to fully understand how domain-specific skills in dance interact with general creative abilities (Teng et al., 2021). This deeper exploration could reveal important insights into the complexities of creativity, both in dance and across various fields. Although the cross-sectional design may not be ideal for determining the specific duration and intensity of training required to manifest functional connectivity benefits for stimulating creative thinking, the findings could provide insights into the neurological basis for the positive effects of neuroplastic reorganization through dance. This non-pharmacological intervention may enhance motor and cognitive abilities in individuals with neurological diseases (Bek et al., 2022; Wu et al., 2022; Meulenberg et al., 2023).

## 5 Conclusion

Long-term dance training strengthens the synergy between metacognitive abilities and motor skills, as reflected in the enhanced FC between NCM and NCD, which is linked to higher levels of creative originality. Although such nuanced neural reorganization and neurodynamic plasticity can be observable without marked shifts in overall ATTA creativity performance, this adaptable FC between NCM and NCD may fine-tune a dancer’s originality, providing a natural advantage in the seamless integration of creative cognitive activities, including mind-wandering and self-reflection. Our study suggests that the consolidation of the NCM-NCD FC as shaped by domain-specific training can inform general creativity.

## Data availability statement

The raw data supporting the conclusions of this article will be made available by the authors, without undue reservation.

## Ethics statement

The studies involving humans were approved by the Institutional Review Board of Taipei Veterans General Hospital. The studies were conducted in accordance with the local legislation and institutional requirements. The participants provided their written informed consent to participate in this study.

## Author contributions

C-JY: Conceptualization, Formal analysis, Investigation, Methodology, Validation, Visualization, Writing – original draft, Writing – review & editing. H-YY: Funding acquisition, Resources, Writing – review & editing. T-YH: Investigation, Writing – review & editing. L-KC: Investigation, Writing – review & editing. W-CL: Investigation, Methodology, Writing – review & editing. T-CY: Funding acquisition, Methodology, Writing – review & editing. L-FC: Funding acquisition, Methodology, Writing – review & editing. J-CH: Conceptualization, Funding acquisition, Methodology, Project administration, Resources, Supervision, Writing – review & editing.
